# 367. Safety and Immunogenicity of a 50-μg mRNA-1273 Vaccine Booster for Severe Acute Respiratory Syndrome Coronavirus-2 (SARS-CoV-2) in Adolescents

**DOI:** 10.1093/ofid/ofad500.437

**Published:** 2023-11-27

**Authors:** Amparo Figueroa, Kashif Ali, Gary Berman, Honghong Zhou, Weiping Deng, Monali Patel, Bethany Girard, Anne Yeakey, Karen Slobod, Frances Priddy, Jacqueline Miller, Rituparna Das

**Affiliations:** Moderna, Inc., Cambridge, Massachusetts; Kool Kids Pediatrics, DM Clinical Research, Houston, Texas; Clinical Research Institute, Allergy and Immunology, Minneapolis, Minnesota; Moderna, Inc., Cambridge, Massachusetts; Moderna, Inc., Cambridge, Massachusetts; Moderna, Inc., Cambridge, Massachusetts; Moderna, Inc., Cambridge, Massachusetts; Moderna, Inc., Cambridge, Massachusetts; Moderna, Inc., Cambridge, Massachusetts; Moderna, Inc., Cambridge, Massachusetts; Moderna, Inc., Cambridge, Massachusetts; Moderna, Inc., Cambridge, Massachusetts

## Abstract

**Background:**

Increased community COVID-19 cases prompted the clinical evaluation of an mRNA-1273 booster dose (BD) in TeenCOVE adolescent participants (12-17 years) who received a 2-dose mRNA-1273 primary series. At ≥5 months after dose 2 (coinciding with the omicron wave peak in Jan 2022), TeenCOVE participants were offered an optional 50-µg mRNA-1273 BD. Here, we inferred BD effectiveness in adolescents by demonstrating non-inferiority (NI) of neutralizing antibody (nAb) responses post-BD vs young adults (18-25 years) post-dose 2 of 100-μg mRNA-1273 primary series in the pivotal phase 3 COVE study, where efficacy was established.

**Methods:**

Up to 6 months post-BD, 1405 participants were monitored for COVID-19 and safety (solicited adverse reactions [≤7 days post-BD]; unsolicited AEs [≤28 days post-BD]; and medically attended, serious [SAEs], of special interest [AESI], or leading to discontinuation [throughout study]). At Day 29 post-BD, nAb geometric mean concentrations (GMCs) were measured against ancestral D614G SARS-CoV-2 spike protein. Binding antibodies (bAbs) against spike protein of ancestral strain or alpha, beta, delta, and gamma variants were measured.

**Results:**

An mRNA-1273 BD was generally well-tolerated; reactogenicity profiles were consistent to the phase 3 COVE study in young adults. There were no severe COVID-19 cases, deaths, or investigator-reported vaccine-related SAEs or AESIs. In pre-booster SARS-CoV-2 negative participants, the ratio of adolescent (n=264) BD-Day 29 GMC (7102; 95% CI, 6553.2-7696.8) to young adult (n=294) Day 57 GMC (1400.4; 1272.7-1541.0) was 5.1 (4.5-5.7), meeting NI criterion for GMR (**Fig 1; Table 1**). The group difference in seroresponse rate (SRR) between adolescents and young adults was 0.7% (95% CI, -0.8 to 2.4), meeting NI criterion for SRR difference (**Table 1**). Robust bAb responses were observed, including against variants.

**Figure:**
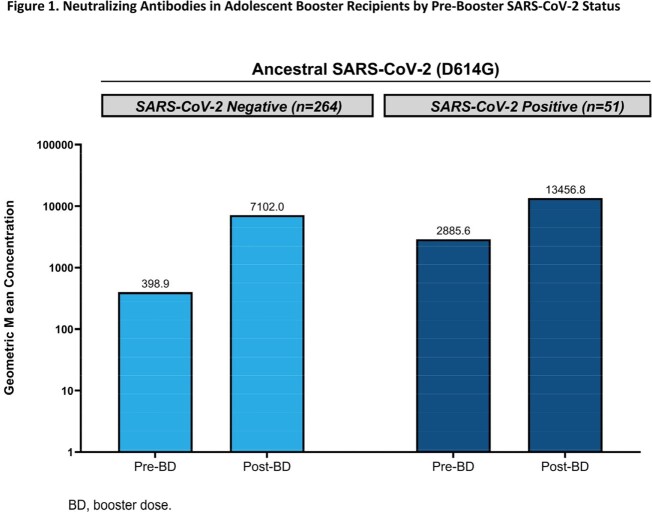

**Figure:**
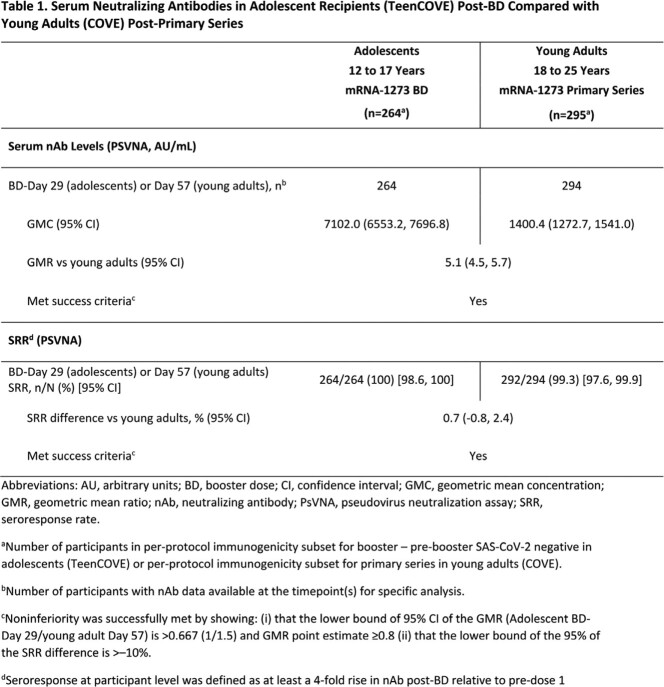

**Conclusion:**

Effectiveness of an mRNA-1273 BD against COVID-19 in adolescents was inferred by successful immunobridging to young adults in the pivotal phase 3 trial. The benefits of variant-containing mRNA-1273 boosters demonstrated in adults is also anticipated to be conferred to adolescents. The overall benefit-risk profile of an mRNA-1273 BD is favorable in adolescents.

**Disclosures:**

**Amparo Figueroa, MD, MPH**, Moderna, Inc.: salary|Moderna, Inc.: Stocks/Bonds **Gary Berman, MD**, Moderna, Inc.: Grant/Research Support **Honghong Zhou, Ph.D.**, Moderna, Inc.: salary|Moderna, Inc.: Stocks/Bonds **Weiping Deng, PhD**, Moderna, Inc.: salary|Moderna, Inc.: Stocks/Bonds **Monali Patel, MS**, Moderna, Inc.: salary|Moderna, Inc.: Stocks/Bonds **Bethany Girard, Ph.D.**, Moderna, Inc.: salary|Moderna, Inc.: Stocks/Bonds **Anne Yeakey, MD**, Moderna, Inc.: Advisor/Consultant **Karen Slobod, MD**, Moderna, Inc.: Advisor/Consultant **Frances Priddy, MD, MPH**, Moderna, Inc.: Salary|Moderna, Inc.: Stocks/Bonds **Jacqueline Miller, MD**, Moderna, Inc.: salary|Moderna, Inc.: Stocks/Bonds **Rituparna Das, M.D.**, Moderna, Inc.: Salary|Moderna, Inc.: Stocks/Bonds

